# Lower Selenoprotein P Is Independently Associated with Peripheral Arterial Disease in Peritoneal Dialysis

**DOI:** 10.3390/diagnostics16030375

**Published:** 2026-01-23

**Authors:** I-Min Su, Chung-Jen Lee, Chiu-Huang Kuo, Chih-Hsien Wang, Bang-Gee Hsu

**Affiliations:** 1 Institute of Medical Science, Tzu Chi University, Hualien 97004, Taiwan; 2 Department of Anesthesiology, Dalin Tzu Chi Hospital, Buddhist Tzu Chi Medical Foundation, Chiayi 62247, Taiwan; 3 School of Medicine, Tzu Chi University, Hualien 97004, Taiwan; 4 Department of Nursing, Tzu Chi University, Hualien 97004, Taiwan; 5 Division of Nephrology, Hualien Tzu Chi Hospital, Buddhist Tzu Chi Medical Foundation, Hualien 97004, Taiwan; 6 School of Post-Baccalaureate Chinese Medicine, Tzu Chi University, Hualien 97004, Taiwan

**Keywords:** peripheral arterial disease, ankle–brachial index, peritoneal dialysis, selenoprotein P, C-reactive protein

## Abstract

**Background/Objectives**: Peripheral arterial disease (PAD) is a common yet often unrecognized complication in patients receiving peritoneal dialysis (PD). Considering that ankle–brachial index (ABI) can be difficult to interpret in this population, additional vascular biomarkers are needed. Selenoprotein P (SePP) is a major selenium transport protein with antioxidant and metabolic regulatory functions and may reflect vascular stress relevant to PAD. We investigated the association of circulating SePP levels with ABI-defined PAD in patients on PD. **Methods**: In this cross-sectional analysis of 98 patients on PD, ABI was assessed using an automated oscillometric device, and ABI < 0.9 was defined as ABI-defined PAD. Serum SePP levels were measured using enzyme-linked immunosorbent assay. **Results**: ABI-defined PAD was identified in 20 patients (20.4%). Compared with patients with normal ABI, those with ABI-defined PAD were older (*p* = 0.014) and had significantly higher prevalence of diabetes mellitus (*p* = 0.033), longer PD vintage (*p* = 0.036), higher fasting glucose (*p* = 0.005) and C-reactive protein (*p* = 0.003) levels, and lower SePP concentrations (*p* < 0.001). Low SePP level remained independently associated with ABI-defined PAD after multivariate adjustment (odds ratio 0.930, 95% confidence interval 0.771–0.997; *p* = 0.032) and consistently across reinforced bootstrap resampling. SePP correlated positively with ABI on the left (*p* = 0.001) and right (*p* = 0.002) sides. **Conclusions**: Among patients undergoing PD, a low serum SePP level was independently associated with ABI-defined PAD and positively associated with ABI, suggesting that SePP may serve as an associative biomarker reflecting vascular vulnerability rather than a diagnostic indicator in this population.

## 1. Introduction

Peripheral arterial disease (PAD) represents a major clinical manifestation of systemic atherosclerosis and is disproportionately prevalent among individuals with chronic kidney disease (CKD) and end-stage kidney disease (ESKD). Epidemiologic evidence indicates that patients with advanced kidney dysfunction develop PAD at rates that far exceed those in the general population, with dialysis cohorts exhibiting a four- to sixfold higher prevalence and substantially increased risks of limb ischemia, amputation, and cardiovascular mortality [1]. Large-scale analyses of inpatients further demonstrated that those who had ESKD and PAD had relatively long admissions, high healthcare costs, and significantly elevated in-hospital mortality, underscoring the considerable clinical burden when these conditions coexist [2]. Although much of the existing literature focused on hemodialysis, patients undergoing peritoneal dialysis (PD) share a comparable risk profile, because several major PAD contributors, including diabetes, hypertension, dyslipidemia, chronic inflammation, oxidative stress, and CKD mineral bone disorder, are also prevalent in PD populations [3]. Additional PD-specific factors, such as hypoalbuminemia, protein loss, malnutrition, and persistent inflammation, may further exacerbate endothelial dysfunction, vascular calcification, and impaired microvascular integrity, thereby increasing susceptibility to lower extremity ischemia [4].

Given the often silent or atypical presentation of PAD in patients with CKD and on PD, timely recognition remains challenging. The ankle–brachial index (ABI) is a widely used, noninvasive, and inexpensive method for assessing lower limb perfusion, with demonstrated strong predictive value for cardiovascular events and mortality in both community and dialysis cohorts [5]. However, ABI reflects a hemodynamic surrogate rather than a definitive anatomic obstruction and does not fully capture clinically or imaging-confirmed PAD, particularly in patients with CKD and PD. Moreover, ABI interpretation in patients on dialysis requires caution, because medial arterial calcification and increased arterial stiffness, which are common in CKD, may lead to falsely elevated values and underdiagnosis of PAD [6]. Despite these limitations, a low ABI remains a robust indicator of PAD severity and portends worse survival among patients receiving long-term dialysis [7]. Consequently, PAD in patients on PD is frequently underrecognized but is clinically consequential, contributing to poor wound healing, infection, limb loss, and excess cardiovascular mortality.

Selenoprotein P (SePP) is the major selenium-transporting glycoprotein in circulation that plays a central role in systemic selenium homeostasis and antioxidant defense. SePP is predominantly synthesized in the liver and is responsible for delivering selenium to peripheral tissues. Impaired SePP production reduces selenium bioavailability, disrupts redox balance, and increases susceptibility to oxidative injury, all of which are closely linked with atherosclerotic vascular disease [8,9]. In addition to its transport function, SePP interacts with vascular endothelial proteoglycans through its heparin-binding domain, suggesting its localized role in modulating vascular redox activity [10]. Recent evidence has further identified SePP as a hepatokine capable of influencing metabolic signaling. In particular, elevated SePP was found to suppress adenosine monophosphate-activated protein kinase (AMPK) activation and promote insulin resistance, which are associated with endothelial dysfunction, inflammation, and accelerated vascular injury [11,12]. Moreover, genetic studies have demonstrated associations between SEPP1 variants, altered SePP isoform distribution, reduced selenium bioavailability, and the coexistence of PAD in patients with aortic disease [10]. Collectively, these findings support SePP as a biologically plausible and potentially relevant factor involved in vascular homeostasis and PAD susceptibility [13].

Despite these insights, the clinical significance of circulating SePP in PAD has not been established in PD populations. Existing research has largely examined selenium deficiency in CKD or explored the metabolic effects of SePP in nonuremic settings, and data on direct evaluation of SePP in relation to lower extremity arterial disease among patients on dialysis remain scarce. Moreover, it is unclear whether SePP level reflects vascular vulnerability beyond traditional cardiovascular and inherent PD-related risk factors, including chronic inflammation, oxidative stress, and protein loss. To address this gap, this study investigated the association between serum SePP concentrations and ABI-defined PAD in individuals undergoing maintenance PD. We further examined whether lower SePP levels are independently associated with ABI-defined PAD after adjustment for conventional cardiovascular and dialysis-specific risk factors.

## 2. Materials and Methods

### 2.1. Ethics and Study Participants

This cross-sectional study consecutively enrolled individuals who had been on PD for more than 6 months due to ESKD at Hualien Tzu Chi Hospital, Taiwan, from 1 February 2020, to 31 May 2020. The study protocol received approval from the Institutional Review Board of Hualien Tzu Chi Hospital (approval number: 108-219-A), and written informed consent was obtained from all participants. No compensation was provided. Patients were excluded if they had acute or active infections, decompensated heart failure, recent acute coronary syndrome or stroke, known malignancies, prior lower limb amputation, or an ABI > 1.3. Clinical parameters related to solute removal (i.e., weekly and peritoneal fractional clearance index for urea (Kt/V) and total and peritoneal creatinine clearance) were retrieved from electronic medical records. Diabetes mellitus (DM) was defined as a fasting plasma glucose level of ≥126 mg/dL or ongoing use of antidiabetic medications. Hypertension was defined as systolic blood pressure ≥140 mmHg, diastolic pressure ≥90 mmHg, or use of antihypertensive agents within 2 weeks prior to enrollment.

### 2.2. Anthropometric Measurements

All anthropometric assessments were conducted with the participants wearing light indoor clothing. Height and body weight were measured three times each, and the mean values were used in the analyses. Standing height was assessed using a stadiometer (H910; Nagata Scale Co., Tainan, Taiwan), and weight was determined using a calibrated digital scale (FM-200; Hostart Co., New Taipei City, Taiwan). Body mass index was calculated as weight divided by height squared (kg/m^2^).

### 2.3. Biochemical Analyses

Blood samples were collected after an overnight fast of at least 8 h and prior to the first daytime PD exchange. Venous blood (5 mL) was drawn into two tubes. An ethylenediaminetetraacetic acid (EDTA) containing tube was used for hemoglobin analysis using a hematology analyzer (Sysmex XS-1000i; Sysmex America, Mundelein, IL, USA). The remaining sample was centrifuged at 3000× *g* for 10 min, and serum was stored at 4 °C until testing. Serum biochemical parameters, including total cholesterol, triglycerides, glucose, albumin, blood urea nitrogen, creatinine, calcium, phosphorus, and C-reactive protein (CRP), were measured using an automated analyzer (Siemens Advia 1800; Siemens Healthcare, Erlangen, Germany). Enzyme-linked immunosorbent assays were used to determine concentrations of intact parathyroid hormone (iPTH) (NM59041, IBL International, Hamburg, Germany) and SePP (CSB-EL021018HU, Cosmo Bio USA, Carlsbad, CA, USA). The respective intra- and interassay coefficients of variation were 3.6% and 2.8% for iPTH and 3.2% and 5.4% for SePP.

### 2.4. Ankle-Brachial Index Assessment

ABI measurements were obtained using an automated oscillometric device (VaSera VS-1000; Fukuda Denshi, Tokyo, Japan). While supine, participants underwent three repeated measurements of systolic pressures on both brachial arteries and both ankles (dorsalis pedis and posterior tibial arteries). The ABI for each leg was calculated by dividing the highest ankle systolic pressure by the highest brachial systolic pressure. The average of three consecutive ABI measurements was used for analysis. Electrocardiographic monitoring was maintained for approximately 15 min during assessment. The device was calibrated in accordance with manufacturer recommendations, and all cuffs were certified and regularly inspected. PAD was defined as an ABI < 0.9 in either limb (ABI-defined PAD), which serves as a noninvasive surrogate marker of lower extremity arterial disease rather than a clinical or imaging-based diagnosis [14].

### 2.5. Statistical Analysis

The required sample size was estimated a priori, indicating that a minimum of 85 participants would be needed to achieve 80% statistical power (ɑ = 0.05) to detect a correlation coefficient of 0.30 between SePP levels and ABI values. Continuous variables were assessed for normality and presented as mean ± standard deviation or median with interquartile range, as appropriate. Group differences were evaluated using Student’s *t*-test or Mann–Whitney U test. Categorical variables were reported as counts with percentages and compared using chi-square analysis. Variables exhibiting skewed distributions, including PD vintage, fasting glucose, iPTH, CRP, and total creatinine clearance, were logarithmically transformed (log−) before correlation analyses and regression modeling. Multivariate logistic regression analysis was performed to identify factors associated with ABI-defined PAD, adjusting for the following clinical variables that exhibited significant differences between patients with and without ABI-defined PAD: PD vintage, age, CRP, fasting glucose, SePP, and diagnosis of DM. To further examine model stability, multivariate logistic regression was conducted with 1000 bootstrap resamples using bias-corrected and accelerated (BCa) confidence intervals. Model calibration was evaluated using the Hosmer–Lemeshow goodness-of-fit test, calibration intercept, calibration slope, and Brier score. A decision curve analysis was used to quantify the clinical utility of SePP-based prediction. Spearman’s rank correlation coefficients were calculated to explore associations among ABI values, SePP, and clinical parameters. To evaluate the diagnostic performance of serum SePP for ABI-defined PAD, receiver operating characteristic (ROC) curves were generated, and the area under the curve (AUC) was calculated (MedCalc Software Ltd., version 22.019, Ostend, Belgium). All analyses except ROC curves were performed using Statistical Package for the Social Sciences version 25.0 (IBM Corp., Armonk, NY, USA). Statistical significance was defined as *p* < 0.05.

## 3. Results

### 3.1. Baseline Characteristics

The clinical characteristics of the study population are summarized in Table 1. Of 98 patients on PD, 20 (20.4%) had ABI-defined PAD. Compared with patients with normal ABI group, those with low ABI group were significantly older (*p* = 0.014); had significantly longer PD vintage (*p* = 0.036); had similar body mass index, blood pressure, lipid parameters, hemoglobin, calcium, phosphorus, and dialysis adequacy metrics; had significantly higher levels of fasting glucose (*p* = 0.005) and CRP (*p* = 0.003); had significantly higher prevalence of DM (*p* = 0.033); and had significantly lower SePP levels (*p* < 0.001).

### 3.2. Factors Associated with Peripheral Artery Disease

Variables with *p* < 0.05 in the comparison of the normal and low ABI groups were included in a multivariate logistic regression model, with adjustments for age, DM, PD vintage, CRP, fasting glucose, and SePP. As shown in Table 2, multivariate logistic regression analysis revealed that ABI-defined PAD was independently associated with each 0.1 mg/dL increase in serum CRP [odds ratio (OR) 1.188, 95% confidence interval (CI) 1.062–1.329; *p* = 0.003] and serum SePP (OR 0.930, 95% CI 0.870–0.994; *p* = 0.032).

### 3.3. Bootstrap Internal Validation

Bootstrap internal validation (1000 iterations) further supported the robustness of these findings (Table 3). A low SePP level remained significantly associated with and continued to be a significant predictor of ABI-defined PAD (bootstrap B = −0.072; BCa 95% CI, −0.152 to −0.034).

**Figure 1:**
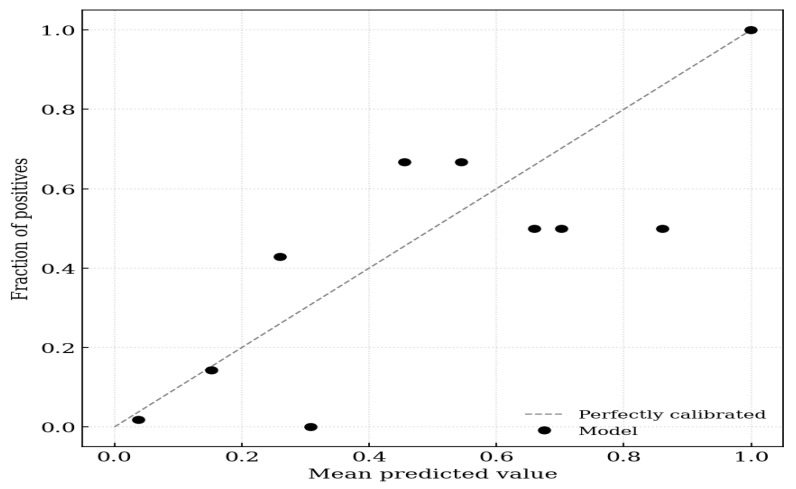
Calibration plot for peripheral arterial disease. Calibration plot by Hosmer–Lemeshow deciles, plot showing observed proportions of peripheral arterial disease (*y*-axis) against model-predicted probabilities (*x*-axis) across 10 deciles of risk. The solid 45° line represents perfect calibration.

Calibration analyses showed good model performance (Hosmer–Lemeshow χ^2^ = 5.429, *p* = 0.711). The calibration intercept was 0.000 (95% CI −0.428 to 0.428), and the calibration slope was 1.000 (95% CI 0.706–1.294), indicating excellent agreement between predicted and observed risk. The Brier score was 0.169, reflecting strong overall predictive accuracy (Figure 1).

### 3.4. Decision Curve Analysis

**Figure 2:**
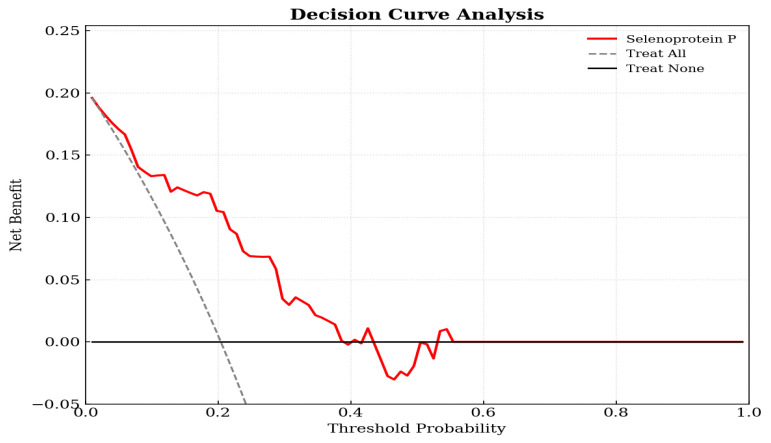
Decision curve analysis for the selenoprotein P-based logistic model predicting ABI-defined peripheral arterial disease. The *y*-axis measures the net benefit. The *x*-axis displays the threshold probability. The red solid line represents the net benefit of using the beta-trace protein prediction model. The gray dashed line represents the assumption that all patients have the outcome (Treat All), while the black horizontal line represents the assumption that no patients have the outcome (Treat None). The decision curve shows that the model provides a higher net benefit across a wide range of threshold probabilities compared to the default strategies.

Decision curve analysis demonstrated that the SePP-based logistic model provided a clear clinical net benefit across a wide range of decision thresholds, compared with treat-all or treat-none strategies (Figure 2). Decision curve analysis results supported the potential clinical utility of the SePP-based logistic model for stratifying ABI-defined PAD risk.

### 3.5. Correlation Analysis

Spearman correlation analysis (Table 4) revealed moderate positive correlations between SePP level and ABI on the left (*r* = 0.336, *p* = 0.001) and right (*r* = 0.314, *p* = 0.002). ABI values were inversely correlated with age, log-PD vintage, log-glucose, and log-CRP (all *p* < 0.05). SePP was found to have negative correlations with log-glucose (*r* = −0.276, *p* = 0.006) and log-CRP (*r* = −0.234, *p* = 0.021), and a positive correlation with residual renal function (urine Clcr; *r* = 0.337, *p* = 0.014), but not with lipid parameters, blood pressure, or dialysis adequacy indices.

### 3.6. Receiver Operating Characteristic Curve Analysis

**Figure 3:**
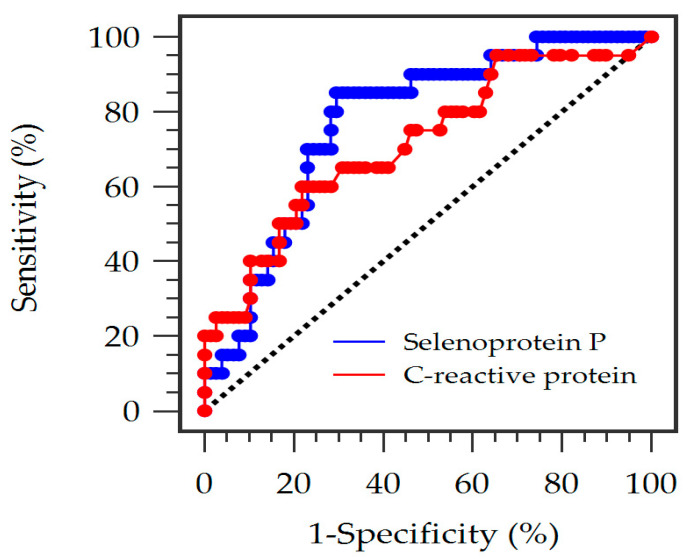
Receiver operating characteristic (ROC) curve analysis to predict arterial disease in 98 peritoneal dialysis patients. The area under the ROC curve (AUC) indicates the diagnostic power of serum selenoprotein P (blue line) and C-reactive protein (red line) levels in predicting peripheral arterial disease in patients undergoing peritoneal dialysis.

ROC curve analysis was performed to compare the diagnostic performance of SePP and CRP for ABI-defined PAD. The ROC analysis shows that serum SePP level has an AUC of 0.774 (95% CI: 0.669–0.880, *p* < 0.001), which is superior to CRP (AUC: 0.716; 95% CI: 0.583–0.850, *p* = 0.0015) in predicting ABI-defined PAD (Figure 3). The optimal cutoff value determined by the Youden index was <20.20 mg/L for SePP, yielding a sensitivity of 85.00% and a specificity of 70.51%. At this threshold, the positive predictive value was 42.50% and the negative predictive value was 94.83%. The optimal cutoff value determined by the Youden index was >0.62 mg/dL for CRP, yielding a sensitivity of 60.00%, a specificity of 78.21%, a positive predictive value of 41.49%, and a negative predictive value of 88.41%. SePP demonstrated a high AUC, suggesting its potential as a diagnostic marker in this population.

## 4. Discussion

In this exploratory cross-sectional study on patients undergoing maintenance PD, low serum SePP level was independently associated with ABI-defined PAD, even after adjustment for age, DM, PD vintage, fasting glucose, and CRP, and this relationship remained robust upon bootstrap internal validation and showed acceptable calibration. SePP correlated positively with both left and right ABI values and urine creatinine clearance and negatively with PD vintage, glycemic indices, and CRP, suggesting that reduced SePP may reflect cumulative vascular injury in this population. Our ROC curve analysis further supports the clinical utility of SePP, demonstrating a notable diagnostic performance for identifying ABI-defined PAD in patients on PD. In addition to SePP, several clinical factors were also associated with ABI-defined PAD, including older age, longer PD vintage, diabetes, elevated fasting glucose, and higher CRP levels, consistent with established mechanisms of vascular injury in CKD and dialysis populations [15,16,17,18,19,20]. These findings highlight the multifactorial nature of PAD in PD and the interplay among metabolic, inflammatory, and dialysis-related contributors.

As the major selenium transport protein, SePP plays an essential role in maintaining systemic antioxidant capacity [21,22]. Reduced SePP may exacerbate oxidative stress and endothelial dysfunction, thereby facilitating atherosclerotic vascular injury [23,24,25]. In patients undergoing PD, chronic inflammation, protein loss, and nutritional deficiencies may further reduce selenium reserves and impair SePP synthesis [26,27,28,29], providing a plausible biological explanation for the observed association between low SePP and ABI-defined PAD. The inverse associations we observed between SePP and CRP, as well as fasting glucose, together with its relation to longer PD vintage, suggest that circulating SePP reflects the cumulative metabolic–inflammatory and nutritional stress that characterizes long-term PD, including protein loss, malnutrition, and persistent low-grade inflammation. In this context, SePP appears to capture a cluster of vascular risk states, oxidative stress burden, chronic inflammation, and impaired nutritional status that are tightly linked to vascular dysfunction and PAD in PD populations. Our results support SePP as a biologically plausible proxy for vascular vulnerability, as it is associated with ABI-defined PAD in patients on PD.

Although previous studies have established the physiological importance of SePP in selenium transport and metabolic regulation [8,9,10,11,30,31], direct evidence linking SePP to PAD remains scarce, and no data specific to PD populations have been available. Given the limitations of ABI in dialysis populations [32], data on the association between circulating SePP and PAD in hemodialysis populations are currently lacking. Most studies in hemodialysis patients have focused on selenium status in relation to inflammation and cardiovascular risk rather than limb ischemia or PAD [33]. Therefore, whether similar associations exist in hemodialysis populations remains to be clarified in future studies. In our study, SePP was primarily evaluated as an associative marker of PAD-related pathophysiology rather than as a fully validated diagnostic test. The ROC-derived cut-off of <20.20 mg/L, with a sensitivity of 85.00% and an NPV of 94.83%, together with good calibration, internal bootstrap validation (1000 iterations), and favorable decision curve analysis, suggests that lower SePP levels are consistently associated with ABI-defined PAD and may have potential utility for ruling out PAD in this population. However, these performance estimates should be interpreted as exploratory and hypothesis-generating rather than as definitive evidence of diagnostic applicability.

This study had several limitations that should be acknowledged when interpreting the findings. First, the cross-sectional design precluded any causal inferences regarding the relationship between SePP levels and ABI-defined PAD; it cannot determine whether reduced SePP directly contributes to vascular pathology or represents a downstream marker of underlying metabolic and inflammatory disturbances. Second, the single-center design and relatively modest sample size of this study may limit the generalizability of our results to broader PD populations and reduce the power to detect more subtle associations. Third, PAD was not confirmed by advanced imaging modalities, such as duplex ultrasonography or computed tomography angiography, but was defined solely by ABI, which reflects a hemodynamic surrogate of lower extremity arterial disease rather than definitive anatomic obstruction. Although ABI is widely recommended for PAD screening, it may underestimate disease severity in patients with CKD and PD due to arterial stiffness and medial vascular calcification, potentially leading to false-negative results. Nevertheless, to minimize misclassification related to noncompressible arteries, patients with ABI > 1.3 were excluded from the present analysis. Fourth, circulating SePP was measured at a single time point, and dynamic changes in selenium status, dietary intake, protein losses, and inflammation over time were not assessed. Additionally, we did not measure other selenium-dependent enzymes or selenium intake, which may have influenced antioxidant capacity and vascular risk. Another limitation is that PD patients with PAD had higher daily peritoneal protein clearance than those without PAD [34]. We did not evaluate peritoneal protein or albumin losses concomitantly with SePP measurements; therefore, we cannot determine whether lower SePP levels simply mirror greater peritoneal protein loss and associated malnutrition, or whether SePP captures additional aspects of vascular and redox imbalance. Finally, although the analysis incorporated several multivariate models adjusting for significant factors, the influence of unmeasured residual confounding variables cannot be excluded. These limitations highlighted the need for larger-scale longitudinal studies to clarify temporal relationships and evaluate the predictive utility of SePP in clinical practice.

## 5. Conclusions

In summary, this study demonstrated that low circulating SePP concentrations were significantly and independently associated with ABI-defined PAD in patients undergoing PD. Given the high prevalence of subclinical PAD and limitations of ABI interpretation in this population, SePP should be interpreted primarily as an associative biomarker rather than as a validated diagnostic test for PAD. While ROC analysis and decision-curve metrics suggest that SePP may have potential for risk stratification, these findings are exploratory and derived from a single-center, cross-sectional cohort with limited sample size. Future longitudinal and interventional studies are warranted to clarify the temporal and mechanistic links among SePP deficiency, selenium homeostasis, and PAD and to determine the effect of correcting selenium or SePP levels on mitigating vascular risk in patients undergoing PD.

## Figures and Tables

**Table 1:** Clinical characteristics of peritoneal dialysis patients according to ankle–brachial index (ABI) category (ABI ≥ 0.9 vs. ABI < 0.9).

Characteristic	All Participants (*n* = 98)	Normal ABI Group (*n* = 78)	Low ABI Group (*n* = 20)	*p* Value
Age (years)	57.55 ± 14.37	55.76 ± 14.46	64.55 ± 11.91	0.014 *
PD vintage (months)	36.24 (18.45–80.79)	33.96 (16.59–69.33)	68.82 (20.61–96.90)	0.036 *
Height (cm)	160.40 ± 8.84	160.13 ± 8.30	161.45 ± 10.87	0.554
Body weight (kg)	64.93 ± 13.91	63.70 ± 13.47	69.73 ± 14.93	0.084
Body mass index (kg/m^2^)	25.11 ± 4.30	24.73 ± 4.31	26.57 ± 4.05	0.088
Left ABI	1.04 ± 0.15	1.07 ± 0.09	0.81 ± 0.14	<0.001 *
Right ABI	1.05 ± 0.16	1.10 ± 0.10	0.82 ± 0.15	<0.001 *
Systolic BP (mmHg)	146.34 ± 17.89	146.81 ± 15.84	144.50 ± 24.74	0.609
Diastolic BP (mmHg)	78.28 ± 8.89	78.42 ± 8.21	77.70 ± 11.40	0.747
Hemoglobin (g/dL)	9.66 ± 1.42	9.56 ± 1.44	10.04 ± 1.31	0.186
Albumin (g/dL)	3.55 ± 0.32	3.52 ± 0.34	3.67 ± 0.25	0.066
Total cholesterol (mg/dL)	163.57 ± 44.22	166.60 ± 44.61	151.75 ± 41.65	0.182
Triglyceride (mg/dL)	134.73 ± 64.85	134.94 ± 58.63	133.95 ± 86.82	0.952
Fasting glucose (mg/dL)	102.00 (91.00–128.25)	99.00 (91.00–112.50)	130.50 (100.00–164.00)	0.005 *
Blood urea nitrogen (mg/dL)	61.73 ± 20.05	62.56 ± 23.46	58.50 ± 15.45	0.465
Creatinine (mg/dL)	10.45 ± 3.31	10.56 ± 3.44	10.02 ± 2.79	0.519
Total calcium (mg/dL)	9.62 ± 0.64	9.57 ± 0.67	9.80 ± 0.48	0.161
Phosphorus (mg/dL)	5.19 ± 1.37	5.19 ± 1.43	5.18 ± 1.15	0.969
iPTH (pg/mL)	186.80 (75.10–446.63)	196.25 (79.45–431.05)	198.20 (52.00–582.78)	0.958
C-reactive protein (mg/dL)	0.29 (0.14–0.75)	0.25 (0.10–0.58)	0.77 (0.25–1.31)	0.003 *
SePP (mg/L)	23.25 ± 11.75	25.52 ± 11.48	14.36 ± 8.12	<0.001 *
Weekly Kt/V	1.94 ± 0.42	1.96 ± 0.43	1.90 ± 0.38	0.591
Peritoneal Kt/V	1.74 ± 0.38	1.74 ± 0.41	1.74 ± 0.29	0.996
Total Clcr (L/week)	54.70 (48.65–64.00)	55.50 (48.33–64.00)	54.05 (51.48–64.15)	0.975
Peritoneal Clcr (L/week)	46.79 ± 11.59	46.02 ± 12.59	49.79 ± 5.69	0.196
Urine Clcr (mL/min)	0.49 (0.00–2.34)	0.72 (0.00–2.57)	0.18 (0.00–1.74)	0.414
Female, *n* (%)	53 (54.1)	43 (55.1)	10 (50.0)	0.681
Diabetes, *n* (%)	43 (43.9)	30 (38.5)	13 (65.0)	0.033 *
Hypertension, *n* (%)	81 (82.7)	65 (83.3)	16 (80.0)	0.725
CAPD model, *n* (%)	36 (36.7)	29 (37.2)	7 (30.0)	0.857
Smoking, *n* (%)	9 (9.2)	7 (9.0)	2 (10.0)	0.887

Values for continuous variables are shown as mean ± standard deviation after analysis by Student’s t-test; variables not normally distributed are shown as median and interquartile range after analysis by the Mann–Whitney U test; values are presented as number (%) and analysis after analysis by the chi-square test. Peritoneal dialysis, PD; ABI, ankle brachial index; BP, blood pressure; iPTH, intact parathyroid hormone; SePP, selenoprotein P; Clcr, clearance of creatinine; Weekly Kt/V, weekly fractional clearance index for urea; CAPD, continuous ambulatory peritoneal dialysis. * Values of *p* < 0.05 were considered statistically significant.

**Table 2:** Multivariate logistic regression assessing clinical predictors of ankle–brachial index (ABI)-defined peripheral arterial disease among 98 peritoneal dialysis patients.

Variables	Odds Ratio	95% CI	*p* Value
Selenoprotein P, 1 mg/L	0.930	0.871–0.994	0.032 *
C-reactive protein, 0.1 mg/dL	1.188	1.062–1.329	0.003 *
Age, 1 year	1.058	0.999–1.120	0.053
Diabetes mellitus, present	2.335	0.454–12.007	0.310
PD vintage, 1 month	1.006	0.995–1.019	0.287
Fasting glucose, 1 mg/dL	1.011	0.992–1.031	0.260

Variables with *p* < 0.05 in the analysis (Table 1) were entered into the model. CI, confidence interval; PD, peritoneal dialysis. * *p* < 0.05 was considered statistically significant.

**Table 3:** Multivariate logistic regression with bootstrap resampling (B = 1000) of the factors correlated to ankle–brachial index (ABI)-defined peripheral arterial disease among peritoneal dialysis patients.

Variables	B	Bca 95% CI	*p* Value
Selenoprotein P, 1 mg/L	−0.072	−0.152, −0.034	0.027
C-reactive protein, 0.1 mg/dL	0.173	−0.047, 0.486	0.019
Age, 1 year	0.056	−0.022, 0.294	0.032
Diabetes, present	0.848	−2.589, 6.545	0.313
PD vintage, 1 month	0.006	−0.016, 0.029	0.332
Fasting glucose, 1 mg/dL	0.011	−0.020, 0.065	0.283

Data were analyzed using multivariable logistic regression with 1000 bootstrap resamples. Candidate variables (diabetes, PD vintage, age, C-reactive protein, fasting glucose, and selenoprotein P) were entered simultaneously into the model. Coefficients (B) are log-odds estimates from the fitted model. Bias-corrected and accelerated (BCa) 95% confidence intervals are based on 1000 bootstrap samples. PD, peritoneal dialysis; BCa, bias-corrected and accelerated; CI, confidence interval.

**Table 4:** Spearman correlation coefficients between ankle–brachial index (ABI), selenoprotein P, and clinical variables in peritoneal dialysis patients.

Variables	Left ABI		Right ABI		Selenoprotein P (mg/L)	
	Spearman’s Correlation Coefficient	*p* Value	Spearman’s Correlation Coefficient	*p* Value	Spearman’s Correlation Coefficient	*p* Value
Age (years)	−0.316	0.002 *	−0.225	0.026 *	−0.163	0.108
Body mass index (kg/m^2^)	−0.193	0.057	−0.110	0.282	−0.110	0.280
Log-PD vintage (months)	−0.204	0.044 *	−0.258	0.010 *	−0.267	0.008 *
Left ABI	—	—	0.802	<0.001 *	0.336	0.001 *
Right ABI	0.802	<0.001 *	—	—	0.314	0.002 *
Selenoprotein P (mg/L)	0.336	0.001 *	0.314	0.002 *	—	—
SBP (mmHg)	0.102	0.318	0.160	0.116	0.076	0.456
DBP (mmHg)	0.144	0.158	0.174	0.087	−0.006	0.950
Hemoglobin (g/dL)	−0.128	0.211	−0.156	0.126	−0.117	0.250
Albumin (g/dL)	−0.130	0.200	−0.114	0.264	−0.186	0.066
Total cholesterol (mg/dL)	0.064	0.528	0.005	0.957	−0.142	0.162
Triglyceride (mg/dL)	−0.115	0.260	−0.093	0.361	0.072	0.482
Log-Glucose (mg/dL)	−0.270	0.007 *	−0.318	0.001 *	−0.276	0.006 *
BUN (mg/dL)	0.054	0.599	0.089	0.383	0.190	0.061
Creatinine (mg/dL)	0.148	0.145	0.143	0.160	−0.051	0.616
Total calcium (mg/dL)	−0.039	0.706	−0.063	0.535	−0.009	0.929
Phosphorus (mg/dL)	−0.022	0.832	−0.016	0.877	0.111	0.275
Log-iPTH (pg/mL)	0.119	0.242	0.096	0.347	0.034	0.737
Log-CRP (mg/L)	−0.315	0.002 *	−0.304	0.002 *	−0.234	0.021 *
Weekly Kt/V	0.071	0.487	−0.015	0.883	0.032	0.756
Peritoneal Kt/V	0.060	0.556	−0.084	0.412	−0.165	0.105
Log-Total Clcr (L/week)	0.055	0.592	0.096	0.348	0.180	0.077
Peritoneal Clcr (L/week)	0.001	0.994	−0.025	0.805	−0.116	0.257
Urine Clcr (mL/min)	0.047	0.736	0.074	0.600	0.337	0.014 *

Data of PD vintage, glucose, iPTH, total Clcr, and C-reactive protein levels showed skewed distribution and, therefore, were log-transformed before analysis. ABI, ankle brachial index; PD, peritoneal dialysis; SBP, systolic blood pressure; DBP, diastolic blood pressure; BUN, blood urea nitrogen; iPTH, intact parathyroid hormone; Weekly Kt/V, weekly fractional clearance index for urea; Clcr, clearance of creatinine. * *p* < 0.05 was considered statistically significant (2-tailed).

## Data Availability

The data presented in this study are available on request from the corresponding author. The data are not publicly available due to privacy and ethical restrictions involving patient confidentiality.

## Abbreviations

The following abbreviations are used in this manuscript:

ABI	Ankle–brachial index
AMPK	Adenosine monophosphate-activated protein kinase
AUC	Area under the curve
BCa	Bias-corrected and accelerated
CKD	Chronic kidney disease
CI	Confidence interval
CRP	C-reactive protein
DM	Diabetes mellitus
EDTA	Ethylenediaminetetraacetic acid
ESRD	End-stage kidney disease
Kt/V	Fractional clearance index for urea
OR	Odds ratios
iPTH	intact parathyroid hormone
PAD	Peripheral arterial disease
PD	Peritoneal dialysis
ROC	Receiver operating characteristic
SePP	Selenoprotein P
